# Plasmonic silver quantum dots coupled with hierarchical TiO_**2**_ nanotube arrays photoelectrodes for efficient visible-light photoelectrocatalytic hydrogen evolution

**DOI:** 10.1038/srep10461

**Published:** 2015-06-12

**Authors:** Zichao Lian, Wenchao Wang, Shuning Xiao, Xin Li, Yingying Cui, Dieqing Zhang, Guisheng Li, Hexing Li

**Affiliations:** 1 Key Laboratory of Resource Chemistry of Ministry of Education, Shanghai Key Laboratory of Rare Earth Functional Materials, College of Life and Environmental Science, Shanghai Normal University, Shanghai, 200234, China (PRC)

## Abstract

A plasmonic Ag/TiO_2_ photocatalytic composite was designed by selecting Ag quantum dots (Ag QDs) to act as a surface plasmon resonance (SPR) photosensitizer for driving the visible-light driven photoelectrocatalytic hydrogen evolution. Vertically oriented hierarchical TiO_2_ nanotube arrays (H-TiO_2_-NTAs) with macroporous structure were prepared through a two-step method based on electrochemical anodization. Subsequently, Ag QDs, with tunable size (1.3-21.0 nm), could be uniformly deposited on the H-TiO_2_ NTAs by current pulsing approach. The unique structure of the as-obtained photoelectrodes greatly improved the photoelectric conversion efficiency. The as-obtained Ag/H-TiO_2_-NTAs exhibited strong visible-light absorption capability, high photocurrent density, and enhanced photoelectrocatalytic (PEC) activity toward photoelectrocatalytic hydrogen evolution under visible-light irradiation (λ > 420 nm). The enhancement in the photoelectric conversion efficiency and activity was ascribed to the synergistic effects of silver and the unique hierarchical structures of TiO_2_ nanotube arrays, strong SPR effect, and anti-shielding effect of ultrafine Ag QDs.

Photoelectrocatalysis (PEC) water splitting is widely recognized as one of the most promising ways to large-scale production of hydrogen in the future^1^, and different materials with kinds of structures such as TiO_2_^2^, Fe_2_O_3_^3^,^4^ etc. have been designed for PEC. Currently, TiO_2_ remains a proper choice as an electrode material for PEC, because of its nontoxicity, photostability and low cost. To date, various TiO_2_ nanostructures, including nanoparticles^5^, nanospheres^6^, nanorods^7^, nanowires^8^, and nanotubes^9^,^10^, have been fabricated for their special physicochemical properties. Among the different geometric shapes, the aligned TiO_2_ nanotube arrays (TiO_2_-NTAs) obtained by the anodization of Ti sheet have aroused considerable scientific interest^11^. TiO_2_-NTAs could offer a large internal surface area, thus supplying several reaction active sites. In addition, the aligned nanotube structure allows for the absorption of light by reducing the loss of light reflection, because photons that enter the nanotubes are less likely to escape due to multiple reflections by the walls^12^. Recently, forming hierarchical TiO_2_ nanotube arrays by coating a photonic crystal layer with larger pore size has been proved an effective way to enhance the light adsorption capability^13^.

However, the practical application of pure TiO_2_-NTAs is still limited by the broad band gap (3.2 eV for anatase) and fast recombination rate of photo-generated electron-hole pairs. Coupling TiO_2_-NTAs with noble metals, including Au^14^, Ag^15^, Cu^16^, Pd^17^, was utilized to overcome the inherent problem of TiO_2_ owing to the high conductivity and SPR effect of noble metals. So far, the nanocrystals of noble metals, especially Ag, have been attracting considerable attention. This is because they could strongly interact with light (in visible or infrared region) due to their extraordinary localized SPR properties and their remarkable photostability^18^,^19^,^20^. Nevertheless, coupling TiO_2_-NTAs with noble metal always suffers from the light shielding effect resulting from the larger nanocrystals size of metal^21^. Thus, it is highly required to obtain ultrafine Ag nanocrystals uniformly coupled TiO_2_-NTAs, while keeping the excellent SPR effect of silver metal. Small size of Ag nanocrystals could be favorable for decreasing the photo-generated electron transfer distance and accelerating the electron transfer rate, thus the SPR-induced photo-electron transfer efficiency would be greatly enhanced^17^. Furthermore, the Ag nanocrystals were proved to be able to possess strong SPR effect even its size was decreased to below 2 nm^22^.

Herein, highly dispersed ultrafine Ag nanocrystals were uniformly loaded on the pore-wall surface of the hierarchical TiO_2_ nanotube arrays *via* pulse electro-deposition route^23^. The average size of Ag nanocrystals could be precisely controlled from 1.3 nm to 21.0 nm through carefully controlling the pulse electro-deposition time. Thus, the as-formed ultrafine Ag QDs enhanced hierarchical TiO_2_-NTAs film was utilized as a working electrode for the photoelectrocatalytic hydrogen evolution under visible-light irradiation, with the aid of a small bias voltage of 0.7 V *vs* a saturated calomel electrode (SCE). The results indicated that loading Ag nanocrystals could greatly enhance the visible-light driven performance in photoelectrocatalytic hydrogen evolution owing to the strong SPR effect of Ag and free light-shielding. The optimal size of Ag coupled with TiO_2_ TNAs was about 2.5 nm, delivering for the highest photoelectrocatalytic H_2_ evolution rate of about 124.4 μmol*cm^−2^*h^−1^. Such Ag nanocrystals enhanced hierarchical TiO_2_-NTAs (Ag/H-TiO_2_-NTAs) electrode exhibited excellent photoelectrocatalytic activity toward hydrogen evolution under visible-light irradiation (λ > 420 nm).

## Results

### Characterizations of Ag/H-TiO_2_-NTAs composites

**Figure 1:**
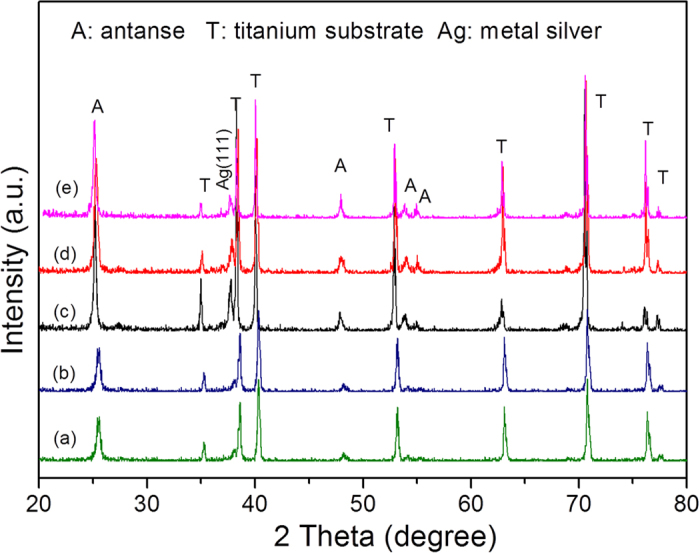
XRD spectra. XRD patterns of (**a**) the as-prepared H-TiO_2_-NTAs, and Ag/H-TiO_2_-NTAs with various pulse electro-deposition time of (**b**) 10 s, (**c**) 20 s, (**d**) 50 s, (**e**) 100 s.

In order to identify the crystal phase of H-TiO_2_-NTAs and Ag/H-TiO_2_-NTAs, the X-ray diffraction (XRD) measurement was used as the results shown in Fig. 1. For the pure H-TiO_2_-NTAs, all diffraction peaks could be indexed to the TiO_2_ anatase phase (JCPDS file no: 21-1272) and the Ti metal phase (JCPDS file no: 44-1294). There were no obvious differences between 10s-Ag/H-TiO_2_-NTAs and pure H-TiO_2_-NTAs. No Ag diffraction peaks could be clearly identified, and it may be attributed to the ultrafine size, high dispersity, and low loaded content of Ag nanocrystals. To the case of the 20 s, 50 s, 100 s Ag/H-TiO_2_-NTAs samples, there were additional diffraction peaks at 2*θ* values of 37.7 ^o^, which could be attributed to the (111) crystal planes of the cubic Ag phase (JCPDF file no: 65-8428)^24^. It should be noted that the other diffraction peaks ascribed to cubic Ag phase were not clearly distinguished because they were overlapped with those of anatase TiO_2_ and pure Ti metal. The XRD results indicated that the Ag/TiO_2_ composite electrodes prepared by the pulse electro-deposition method were composed of Ag nanocrystals and crystalline TiO_2_ anatase.

### FESEM, TEM images and size distribution

**Figure 2:**
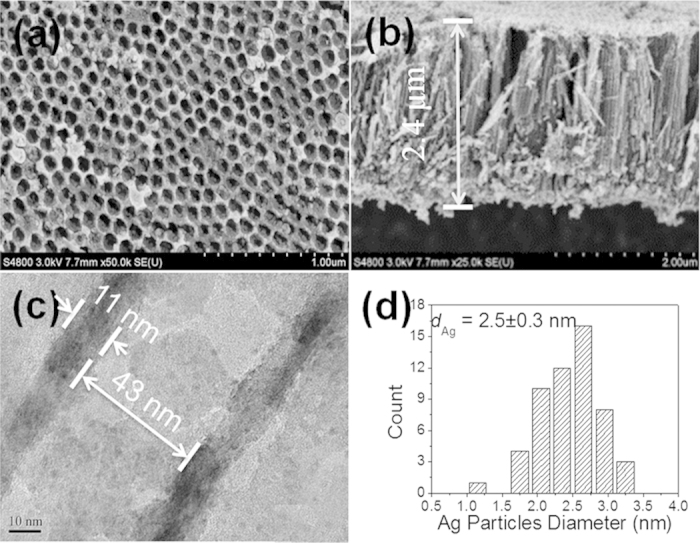
FESEM, TEM images and size distribution. Top view of FESEM image (**a**) cross-section view FESEM image (**b**) of H-TiO_2_-NTAs, (**c**) TEM image of 20s-Ag/H-TiO_2_-NTAs, and (**d**) size distribution of Ag QDs located on the pore wall of 20s-Ag/H-TiO_2_-NTAs.

**Figure 3:**
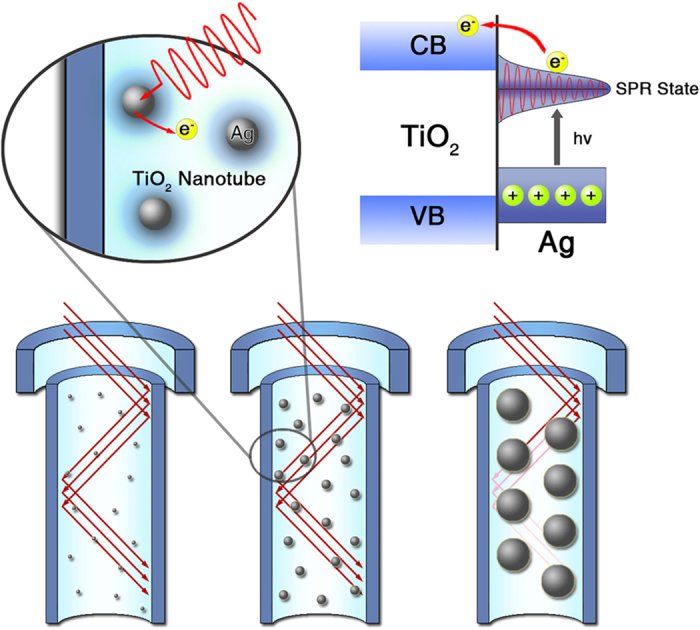
Schematic diagram. The light penetration mechanism in the Ag nanocrytals loaded H-TiO_2_-NTAs.

Figure 2a presents a top view field emission scanning electron microscope (FESEM) image of the as-prepared H-TiO_2_-NTAs. A unique top layer with a periodically porous structure could be clearly observed. The average diameter of the pores was 70 ± 2.0 nm, and the thickness of the walls was 22 ± 1.0 nm. The thickness of the bottom TiO_2_-NTA layer was about 2.4 μm, as shown in Fig. 2b. It should be noted that the length of the TiO_2_-NTAs in this study was rationally chosen, as it has been reported that about 2.0 μm was the maximum penetration depth of the incident light in TiO_2_-NTAs^13^. Further increasing the tube length would improve its electronic resistance, inhibiting the photo-generated transmission located in the TiO_2_-NTAs. A cross-section FESEM view of the H-TiO_2_-NTAs (Fig. 2b) indicated a uniformly non-flat, concave-like top layer, which was closely connected with the bottom TiO_2_-NTAs. The concaves in the top layer were expected to work as nanomirrors for light reflection and scattering^13^. From Fig. 2c,d, one could clearly see that silver QDs were uniformly deposited on the surface of the pore wall of H-TiO_2_-NTAs with an average size about 2.5 nm *via* the proposed pulse electro-deposition route. The crystal lattice of Ag and TiO_2_ can be clearly seen shown in Fig. S1. The lattice space of lattice facet with Ag (111) was 0.238 nm, and the lattice spacing of 0.352 nm was assigned to the (101) facet of TiO_2_. Besides, it can also be observed that the inner diameter of the nanotubes was 43 ± 1.0 nm, and the thickness of the TiO_2_ nanotubes was 11 ± 2.0 nm. Both of the values were much lower than those of the porous surface layer of TiO_2_. As shown in Fig. S2, the size of the loaded Ag nanocrystals could be tunable from about 1.3 nm to 21.0 nm by increasing the pulse electro-deposition time from 10 s to 100 s. The space of the inner nanotubes were almost totally complete occupied by the large Ag nanocrystals upon choosing 100 s electro-deposition time. Such blocking effect resulting from the over growth of Ag nanocrystals will not allow the light to penetrate into the TiO_2_ nanotubes *via* multiple-reflection as shown in Fig. 3. It was obvious that Ag, as photosensitizer, could exhibit SPR effects when it was irradiated with visible-light (490 nm > λ > 390 nm)^19^,^25^. With the aid of SPR effect, hot electrons were able to transfer from Ag nanocrystals into the conduction band of TiO_2_-NTAs. Meanwhile, the wall of TiO_2_ nanotubes possessed excellent ability to transfer electrons, allowing more electrons to be trapped by protons with the formation of H_2_. Thus, it was reasonable that Ag/H-TiO_2_-NTAs would exhibit enhanced photoelectrocatalytic activities in H_2_ evolution owing to the SPR effect and anti-shielding effect of ultrafine Ag QDs.

### XPS analysis

**Figure 4:**
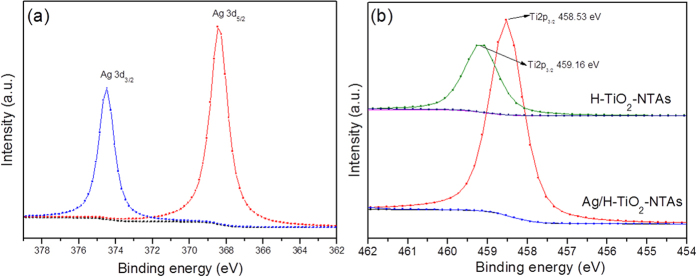
XPS analysis. High resolution XPS of (**a**) Ag 3d of 20s-Ag/H-TiO_2_-NTAs and (**b**) Ti 2p of H-TiO_2_-NTAs and 20s-Ag/H-TiO_2_-NTAs.

In order to acquire in-depth fundamental information on the interaction of Ag with TiO_2_, the X-ray photoelectron spectroscopy (XPS) technique was employed to analyze the specific surface composition and elemental binding energy of H-TiO_2_-NTAs and 20 s Ag/H-TiO_2_-NTAs. Figure 4a exhibited the high-resolution spectrum of Ag 3d from the Ag modified H-TiO_2_ NTAs sample. The Ag 3d_5/2_ core level of 20 s Ag/H-TiO_2_-NTAs could be fitted with a single peak at a binding energy of 368.5 eV, which was attributed to the presence metallic Ag^0^. A significant positive shift of the binding energy for Ag 3d_5/2_ relative to 368.0 eV of the bulk Ag was identified due to the electron transfer from Ag to oxygen vacancies of the TiO_2_.^9^ Such positive shift may be attributed to the low work function of metal Ag. Moreover, a small negative shift of the Ti 2p_3/2_ after loading Ag (shown in Fig. 4b) also revealed the feasibility of the electron transfer between the Ag and H-TiO_2_-NTAs.

### Electrochemical testing and Photoluminescence

**Figure 5:**
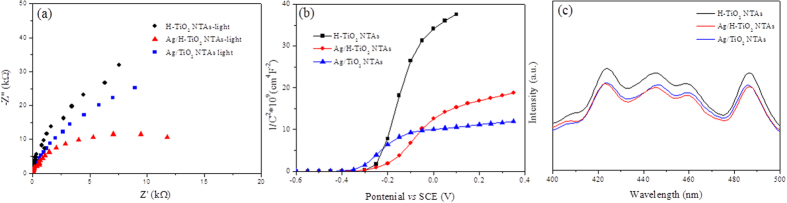
Electrochemical testing and Photoluminescence. (**a**) Nyquist plot of electrochemical impedance spectra, (**b**) Mott-Schottky plots, and (**c**) photoluminescence (PL) spectra with the excited wavelength at 280 nm of H-TiO_2_-NTAs, 20s Ag/H-TiO_2_-NTAs, and 20s Ag/TiO_2_-NTAs.

The interfacial properties between the electrode and the electrolyte were detected by electrochemical impedances spectroscopy (EIS) measurements. A semicircle in the Nyquist plot at high frequency represented the charge-transfer process, while the diameter of the semicircle reflected the charge-transfer resistance (Fig. 5a). It was clear that the arch for Ag/H-TiO_2_-NTAs under visible light (>420 nm) illumination was much smaller than that for H-TiO_2_-NTAs, implying that decoration with Ag QDs could significantly enhance the electron mobility by reducing the recombination of electron-hole pairs. *Via* comparison of the different samples, the arch value for Ag/TiO_2_-NTAs without porous top layer was in the middle among them under visible-light irradiation. It was noted that Ag QDs exhibited an important role in the solution-action process of electron transfer. In addition, the capacitance measurement was performed on the electrode/electrolyte according to the Mott-Schottky equation^26^,
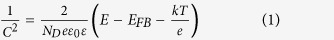
where C is the space charge capacitance in the semiconductor, N_D_ is the electron carrier density, e is the elemental charge value, ε_0_ is the permittivity of the vacuum, ε is the relative permittivity of the semiconductor, E is the applied potential, E_FB_ is the flat band potential, T is temperature, and k is the Boltzmann constant. Figure 5b displays the Mott-Schottky plots of 1/C^2^ as a function of the applied potential, from which the positive slopes (i.e., lines) were observed, suggesting n-type semiconductors. Furthermore, the plots were extrapolated to 1/C^2^ = 0 to estimate the values of E_FB_ at −0.251 V and −0.213 V for H-TiO_2_ NTAs and Ag/H-TiO_2_-NTAs, respectively. A 38 mV increase of E_FB_ implied steeper band bending, forming strong Schottky junction between TiO_2_ and Ag, and thereby facilitating the electron transfer. In addition, the carrier density N_D_ could also be calculated from Fig. 3b by using the following equation^13^:
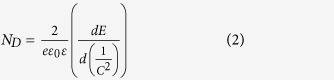
As e = 1.6 × 10^−19^ C, ε_0_ = 8.86 × 10^−12^ F/m, and ε = 48 for anatase TiO_2_, the N_D_ values of H-TiO_2_-NTAs and Ag/H-TiO_2_-NTAs are determined as 1.42 × 10^17^ cm^−3^ and 4.32 × 10^17^ cm^−3^, respectively. Consequently, the higher N_D_ of Ag/ H-TiO_2_-NTAs would result in lower resistance and faster charge transfer rate, leading to enhanced PEC performance.

We noted that the capability of charge separation by Ag QDs could also be verified by the analysis of the photoluminescence (PL) spectra as shown in Fig. 3c. PL measurements were often employed to study surface processes involving electron-hole recombination of TiO_2_. Briefly, after irradiation of the photocatalysts, electron-hole pairs underwent a recombination process, and photons were then emitted, resulting in PL^18^. As shown in Fig. 5c, the peak at 424 nm (corresponding to 2.92 eV) could be attributed to self-trapped excitations located on the TiO_2_ octahedral. The peaks at 446 nm, 459 nm, and 487 nm (corresponding to 2.78 eV, 2.70 eV, and 2.55 eV, respectively) were also observed, which were associated with the oxygen vacancies^27^,^28^,^29^,^30^. The PL intensity for 20 s Ag/H-TiO_2_-NTAs was much lower than that of H-TiO_2_-NTAs, indicating a reduced charge carrier recombination resulting from the formation of Schottky junction between TiO_2_ and Ag nanocrystals^31^. It was also noted that the pure H-TiO_2_-NTAs exhibited a lower intensity of PL compared to that of the Ag loaded traditional TiO_2_ nanotube arrays (20 s Ag/TiO_2_-NTAs). This suggested that the formation of hierarchical structured TiO_2_ nanotubes played an important role for inhibiting the electron-hole recombination.

## Discussion

### Photoelectrochemical properties of the pure and Ag QDs modified H-TiO_2_-NTAs and schematic diagram of SPR charge carrier transfer mechanisms

**Figure 6:**
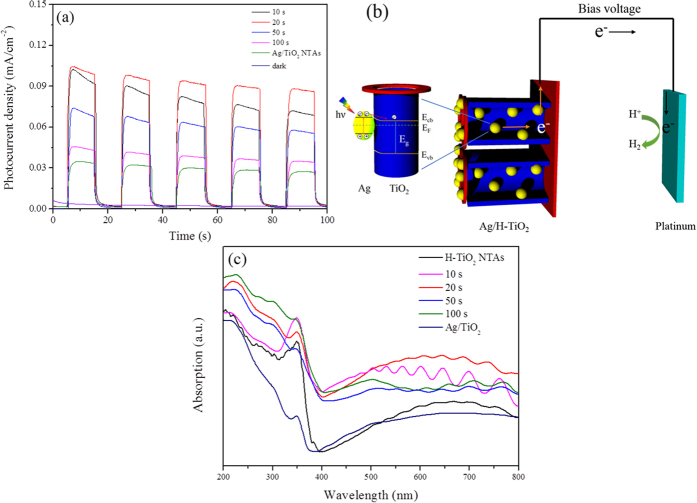
Photoelectrochemical properties of the pure and Ag QDs modified H-TiO_2_-NTAs and schematic diagram of SPR charge carrier transfer mechanisms. (**a**) Photocurrent responses in the light on-off process (0.7 V vs. SCE): under illumination of visible light with wavelength >420 nm with 20 s light on/off cycles. (**b**) SPR charge carrier transfer under visible light irradiation at Ag/H-TiO_2_ interface and the PEC process for H_2_ evolution. (**c**) DRS of H-TiO_2_-NTAs, Ag/TiO_2_ and Ag/H-TiO_2_-NTAs with different deposition time: 10 s, 20 s, 50 s, 100 s.

To evaluate the enhanced PEC performance of the fabricated Ag/H-TiO_2_-NTAs under visible-light irradiation, the transient photocurrent was done to verify the separation of photo-induced charges. Chronoamperometric I-t curves were measured and recorded in Fig. 6a by irradiating the electrodes with visible-light (λ > 420 nm) at a potential of 0.7 V vs SCE. It was surprising that 20 s-Ag/H-TiO_2_-NTAs with hierarchical structures exhibited a very high photocurrent density (0.104 mA/cm^−2^), about 3 times of that (0.034 mA/cm^−2^) of the traditional Ag/TiO_2_-NTAs. Such greatly enhanced photo-response ability could be attributed to its special structure and enhanced SPR intensity. It was also noted that the photocurrent density of Ag/H-TiO_2_-NTAs increased with the pulse deposited time of Ag nanocrystals. Prolonging the deposition time over 20 s could result in the decrease of photocurrent density because of the formation larger size of Ag nanocrystals, which could shield the multi-reflection of light in the hierarchical TiO_2_-NTAs.

As schematically presented in Fig. 6b, Ag QDs, as the sensitizer, were excited under visible-light irradiation. Hot electrons from the SPR of metal Ag, possessed enough energy to overcome the Schottky barrier between the Ag and TiO_2_-NTAs^32^,^33^ and inject into the conduction band of the adjacent H-TiO_2_-NTAs. Meanwhile, these hot electrons would transfer along the wall of TiO_2_-NTAs, which could provide the fast transfer paths. Besides, the ultrafine size of Ag nanocrystals could also be favorable for enhancing the transfer rate of electrons owing to its short electron-migration distance between excited Ag and the wall of TiO_2_-NTAs.

With the aid of external bias voltage, the electrons could transfer from photoanode to cathode (platinum foil), and further react with the H^+^ ions to form H_2_ on the surface of Pt foil. The positive charges formed on Ag QDs possessed certain oxidation ability to participate in oxidation reaction, in which ethylene glycol was transfered to glyoxal, oxalic acid or other by-products. In addition, the optical properties of H-TiO_2_-NTAs and Ag/H-TiO_2_-NTAs samples were investigated by UV-vis diffuse reflectance spectroscopy (DRS). As shown in Fig. 6c, the absorption edge at a wavelength lower than 380 nm could be attributed to the intrinsic band-gap absorption of anatase TiO_2_. The absorption of bare H-TiO_2_-NTAs (Fig. 6c) in the visible-light region could be assigned to the hierarchical ordered nanoarrays. Such ordered TiO_2_ nanoarrays can form the photonic crystals, allowing the TiO_2_ films to adsorb visible-light^13^. However, such adsorbed visible light cannot be utilized to activate the pure TiO_2_ for the formation of electro-hole pairs. Upon loading Ag nanocrystals, Ag/H-TiO_2_-NTAs exhibited similar multi-peaks in the visible-region and a significant enhancement of visible-light absorption due to the SPR absorption of the Ag QDs. Among all these Ag enhanced H-TiO_2_-NTAs samples, 20s Ag/H-TiO_2_-NTAs displayed the highest SPR intensity, indicating that the SPR effect of Ag was well maintained even its size was decreased to about 2.5 nm *via* carefully choosing the pulse electro-deposition route. This was consistent with the measured SPR wavelength of 440 nm of the same-sized Ag^22^ nanocrystals dispersed in solution. And an enhanced visible-light absorption region (wavelength 400-550 nm) was obvious when the Ag NPs size was very small^34^ compared with other samples (in Fig. S2). It should be pointed out that further increasing the size of Ag nanocrystals, *via* extending the pulse electron-deposition time over 20 s, resulted in a decrease of the SPR intensity in Fig. 6c. Such decrease could be ascribed to the light shielding effect of the Ag nanocrystals with larger grain size on the pore wall of TiO_2_ nanotubes.

### Size of Ag QDs and photocatalytic H_2_ production

**Figure 7:**
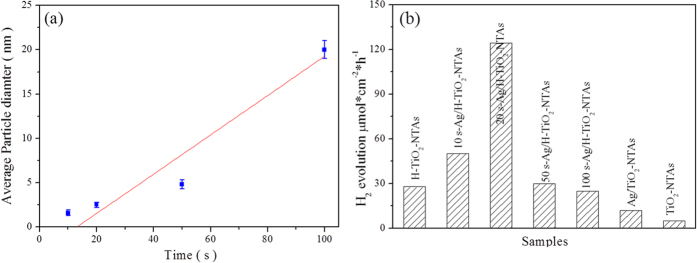
Size of Ag QDs and photocatalytic H_2_ production. (**a**) The size of Ag quantum dots *via* the pulse electron-deposition time; (**b**) the hydrogen evolution rate by using various samples as photoanodes and Pt foil as cathodes, at 0.7 vs. SCE in a PEC cell containing a 2 M ethylene glycol and 0.5 M Na_2_SO_4_ solution under 300 W Xe lamp (>420 nm filter) irradiation.

As shown in Fig. 7a, the size of Ag QDs could be tuned from 1.6 ± 0.3 nm to 20 ± 1.0 nm *via* choosing various pulse-deposition time. Based on the linear fitting equation (y = −2.9 + 0.22x,y represents the size of Ag QDs, x is the deposition time), it was noted that the size of Ag QDs could be controlled by the deposition time consistent with a linear variation. Thus, the pulse deposition method was a suitable route for the fabrication of Ag QDs with controllable size. These results were consistent with the SEM images in Fig. S2. Figure 7b shows the hydrogen generation performance of Ag QDs enhanced hierarchical TiO_2_ nanotubes arrays *via* choosing traditional TiO_2_ nanotubes as standard. The sample of 20 s Ag/H-TiO_2_-NTAs exhibited the highest H_2_ evolution rate (124.5 μmol*cm^−2^*h^−1^) among all these Ag QDs modified hierarchical TiO_2_ nanotube arrays. Also it indicated that the macroporous structure covering on the surface of TiO_2_ nanotubes contributed lots to enhancing the photoelectrocatalytic activity of electrodes compared to the traditional TiO_2_ nanotubes. The amount of hydrogen production exhibited a volcanic pattern from H-TiO_2_-NTAs to the Ag/H-TiO_2_-NTAs with increasing the size of Ag QDs (1.3 nm to 21.0 nm). The relative low H_2_ production rate over 10 s-Ag/H-TiO_2_-NTAs was attributed to the lower loaded amount of Ag. The poor H_2_ production rate over H-TiO_2_-NTAs with large size of Ag QDs may be attributed to the shielding effect toward light of the sizes, which was consistent with the order of light absorption capability in visible-light region as shown in Fig. 6c. It should be pointed out that the H_2_ evolution rate of hierarchical TiO_2_-NTAs, upon being loaded Ag QDs (20 s deposition time), exhibited a significant increase, more than 3.45 times of that of pure hierarchical TiO_2_-NTAs. However, the H_2_ production rate of Ag/TiO_2_-NTAs only exhibited an increase about 1.4 times compared to that of pure TiO_2_-NTAs. These results could further confirm that the hierarchical structure with modification of Ag QDs would enhance the photoelectrocatalytic activity for H_2_ production. It might be attributed to the strong Ag-TiO_2_ interaction and the excellent light-utilization efficiency of Ag QDs and H-TiO_2_-NTAs.

In summary, Ag QDs enhanced hierarchical TiO_2_-NTAs electrode was fabricated for effectively driving H_2_ evolution through photoelectrocatalytic water-splitting under visible-light (λ > 420 nm) irradiation. Ag QDs with various average size (1.3-21.0 nm) could be uniformly deposited on the surface of the TiO_2_ hierarchical nanotubes. Owing to the strong SPR effect and anti-shielding effect of ultrafine Ag QDs, and the unique property of hierarchical structures of TiO_2_ nanotube arrays, the photoelectric conversion efficiency and activity were greatly increased. Especially, the special hierarchical TiO_2_ nanotubes with macroporous layers was proved effective for enhancing the interaction between Ag QDs and TiO_2_, with the formation of high H_2_ evolution activity.

## Methods

### Materials

Titanium sheets (0.3 mm thick, 99.5%) were purchased from Shanghai Right Titanium Industry Co., Ltd. ethylene glycol (EG), ammonium fluoride, silver nitrate, sodium nitrate, sodium sulfate, acetone and ethanol of analytical grade were obtained from Aladdin Company without further purification. All solutions were prepared using deionized (DI) water with a resistivity of 18.2 MΩ cm prepared by Millipore system.

### Preparation of H-NTAs

A pure TiO_2_ nanotube arrays electrode was fabricated by an electrochemical anodic oxidation technique. Prior to anodization, the titanium sheets were rinsed in an ultrasonic bath of acetone, ethanol and distilled water for 15 min successively. The anodization was carried out using a conventional two-electrode system with the Ti sheet as the working electrode and a Pt foil (99.99%, 0.1 mm, 2 cm * 2 cm) as the counter electrode, respectively. All electrolytes consisted of 0.5 wt% NH_4_F in ethylene glycol solution and 2 vol% water. Firstly, the Ti sheet was anodized at 60 V for 1.5 h, then the anodized Ti sheet was ultrasonically removed in DI water. Subsequently, a second anodization of the same Ti sheet was performed at 30 V for 0.5 h. All the anodization processes were carried out at 30 ^o^C. After the two-step anodization, the prepared hierarchical structure TiO_2_-NTAs samples were cleaned with DI water. All the as-anodized samples were crystallized by ambient annealing (500 ^o^C) with a heating and cooling rate of 2 ^o^C min^−1^ for 2 h.

### Preparation of Ag/H-NTAs

**Figure 8:**
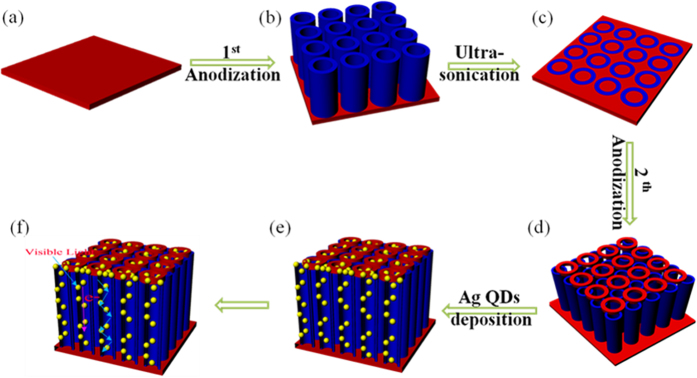
Schematic diagram of the Ag/H-TiO_2_-NTAs fabrication procedure. (**a**) Ti foil, (**b**) first anodized TiO_2_ nanotubes, (**c**) nanoprints left on the Ti foil, (**d**) Second anodized on the TiO_2_ NTAs, (e) Ag QDs deposition on the H-TiO_2_-NTAs *via* a pluses deposition, (**f**) Top larger pore enhancing SPR of Ag QDs and multiple reflections in the H-TiO_2_-NTAs.

A two-electrode setup was used for pulse electrodeposition by using a H-TiO_2_-NTAs electrode as the working electrode, Pt sheet as the counter electrode, 1 mM AgNO_3_ and 10 mM NaNO_3_ as electrolyte solution. For Ag deposition, a current pulsing approach was utilized *via* choosing a cathodic pulse (−25 mA 0.1 s) and a relation time (0 mA, 0.3 s) in 1 mM AgNO_3_ as well as 10 mM NaNO_3_ at room temperature with magnetic string at 200 rpm and different deposited time. The as-obtained products of Ag QDs modified H-TiO_2_ were labeled as t-Ag/H-TiO_2_-NTAs, t represents the time of depositing Ag QDs. All the procedures were showed in Fig. 8. As a reference, the Ag/TiO_2_-NTAs were prepared using an one-step anodization route (30 V, 0.5 h and 30 ^o^C), and followed by the pulse electrodeposition (deposition time: 20 s) with the same method in the preparation of Ag/H-TiO_2_-NTAs as shown in Fig. S3.

### Materials characterizations

X-ray diffraction (XRD) patterns were collected on a Rigacu Dmax-3C with Cu Kα radiation. Selected area electron diffraction (SAED) images and transmission electronic microscopy (TEM) morphologies were recorded on a JEM-2010 instrument. Field emission scanning electron microscopy (FESEM) images were performed on a JEOL JSM-6380LV. The photoluminescence (PL) spectra were recorded on a Varian Cary-Eclipse 500 Raman spectra, and UV-vis diffuse reflectance spectra (UV-vis DRS) were conducted on Dilor Super LabRam II and MC-2530 instruments, respectively. X-ray photoelectron spectroscopy (XPS) analysis was performed on a Perkin-Elmer PHI 5000C ESCA system. All binding energies were calibrated by using the contaminant carbon (C1s = 284.8 eV) as a reference.

### Electrochemical measurements

Photoelectrochemical measurements were carried out in a conventional three-electrode, single-compartment quartz cell on an electrochemical station (CHI 660D). The H-TiO_2_ NTAs electrode and the TiO_2_-NTAs electrode with an active area of ca. 4 cm^2^ served as working electrodes. The counter electrode and the reference electrode were a platinum sheet and saturated calomel electrode (SCE), respectively. A bias voltage of 0.7 V was utilized for driving the photo-generated electrons transfer from the working electrode to the platinum electrode. A 300 W Xe lamp with an ultraviolet filter (λ > 420 nm) used as the visible light source was positioned 10 cm away from the photoelectrochemical cell. A 0.5 M Na_2_SO_4_ aqueous solution was used as the electrolyte. Impedance measurements were performed under illumination (300 W Xe lamp) in 0. 5 M Na_2_SO_4_ solution at open circuit voltage over a frequency range from 10^5^ to 10^−1^ Hz with an AC voltage at 50 mV. The Mott-Schottky plots were obtained at a fixed frequency of 1 KHz to determine the flat-band potential and carrier density.

### Hydrogen evolution measurements

**Figure 9:**
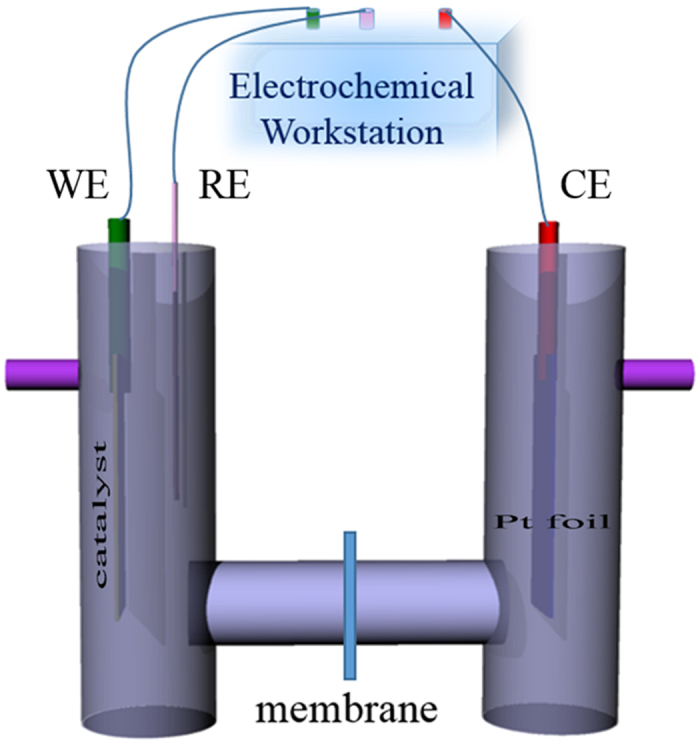
Schematic representation of H_2_ production device. Schematic diagram of the home-made gas generation.

Hydrogen evolution measurements were carried out with a home-made reactor instrument which contains two houses separated by different tubular chambers made by quartz showed in Fig. 9. Such reactor can avoid the mixing of hydrogen generated on the Pt electrode and oxygen (consumed by sanctified agent) generated on the photoanode, and thus the hydrogen and oxygen gases can be separately collected. The home-made reactor device was filled with a 2 M ethylene glycol and 0.5 M Na_2_SO_4_ solution. Ag/H-TiO_2_-NTAs was used as photoanode. SCE electrode was used as reference electrode. Pt foil was used as counter electrode. For testing the amount of the generated H_2_, a 0.5 mL of the gas was sampled intermittently through the septum after 1 h visible-light irradiation, and hydrogen was analyzed by gas chromatograph (GC9800 (N)), Shanghai Ke Chuang Chromatograph Instruments Co. Ltd, China, TCD, with nitrogen as a carrier gas and 5 A molecular sieve columns.

## Additional Information

**How to cite this article**: Lian, Z. *et al.* Plasmonic silver quantum dots coupled with hierarchical TiO_2_ nanotube arrays photoelectrodes for efficient visible-light photoelectrocatalytic hydrogen evolution. *Sci. Rep.* **5**, 10461; doi: 10.1038/srep10461 (2015).

## Supplementary Material

Supporting Information
