# Supplementary material for: Plasmonic silver quantum dots coupled with hierarchical TiO2 nanotube arrays photoelectrodes for efficient visible-light photoelectrocatalytic hydrogen evolution
Source: Sci Rep. 2015 Jun 12;5:10461. doi: 10.1038/srep10461 (PMC4464392; doi:10.1038/srep10461)
Supplement: Supporting Information [file srep10461-s1.doc]

**Supporting Information**

**Plasmonic Silver Quantum Dots Coupled with** **Hierarchical TiO_2_ Nanotube Arrays Photoelectrodes for Efficient Visible-Light Photoelectrocatalytic Hydrogen Evolution**

*Zichao Lian^†^, Wenchao Wang^†^, Shuning Xiao, Xin Li , Yingying Cui, Dieqing Zhang*, Guisheng Li*, Hexing Li*

*Key Laboratory of Resource Chemistry of Ministry of Education, Shanghai Key Laboratory of Rare Earth Functional Materials, College of Life and Environmental Science, Shanghai Normal University, Shanghai, 200234, China (PRC). E-mail: Liguisheng@shnu.edu.cn, Fax: + (86)21-64322272; Tel: + (86)21-64321673.*

**
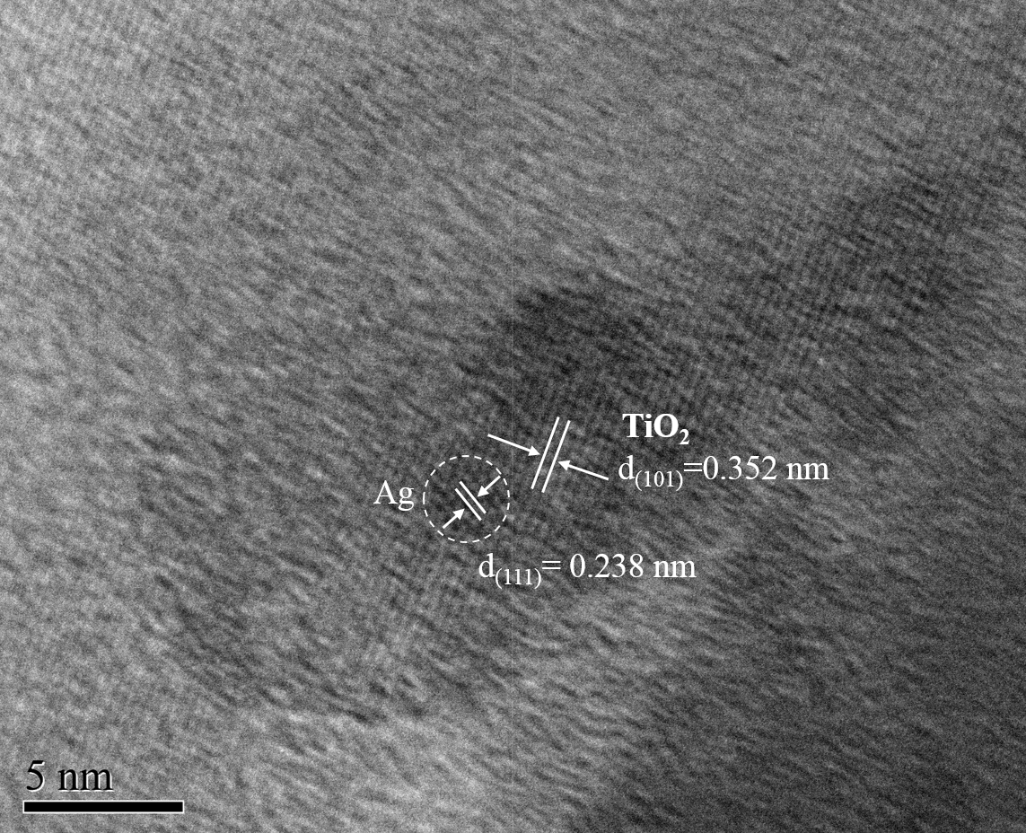
**

**Figure S1.** HR-TEM of 20 s Ag/H-TiO_2_-NTAs.


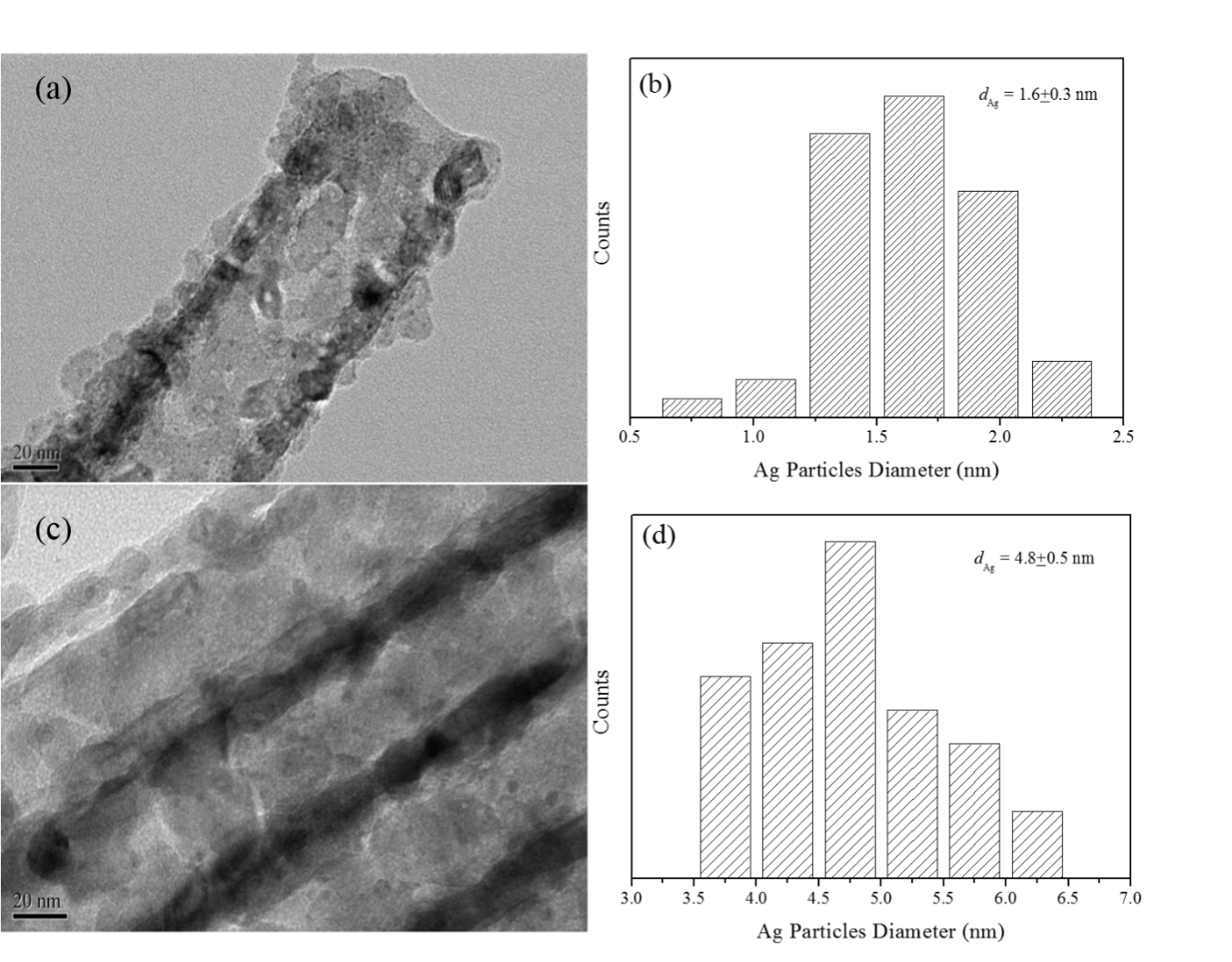

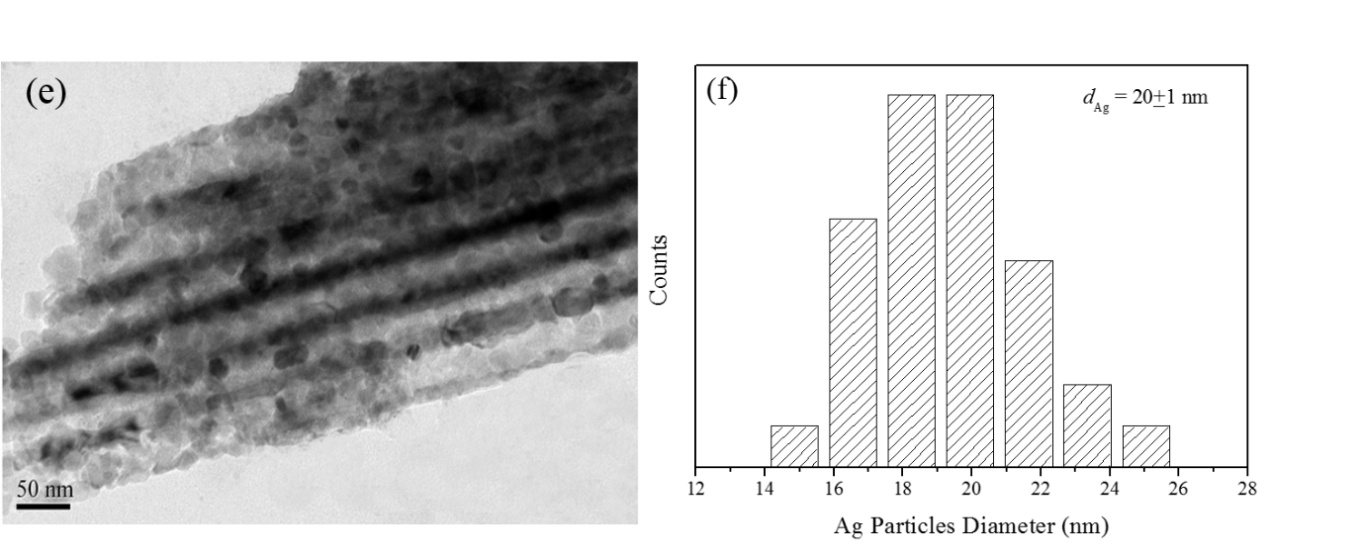


**Figure S2**. TEM of different deposition time of Ag on H-TiO_2_ NTAs (a) 10 s, (c) 50 s, (e) 100 s, and top right inset of Ag particle size distribution.


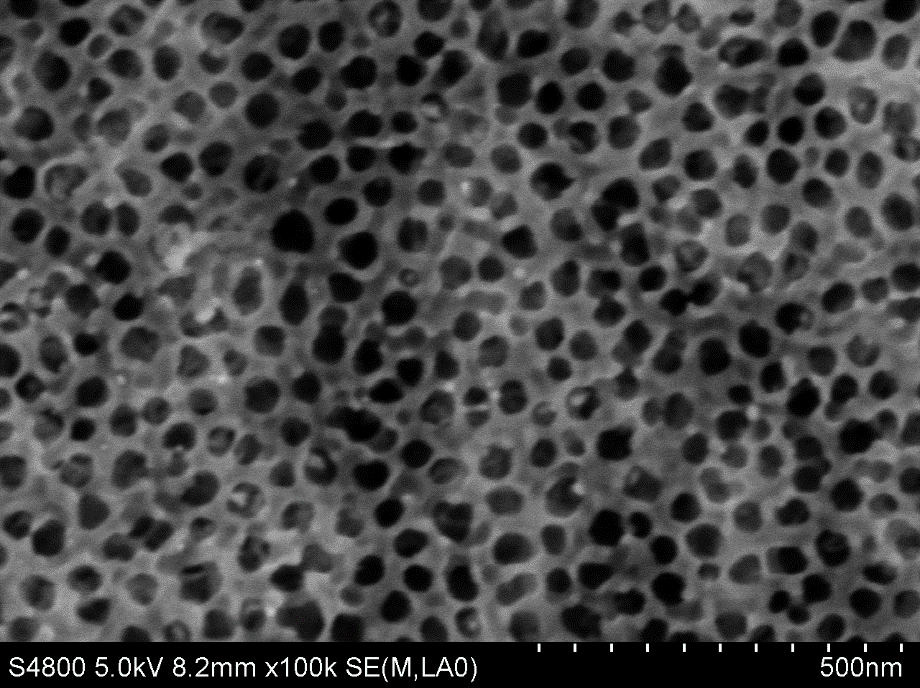


**Figure S3.** The FESEM of Ag/TiO_2_ NTAs (30 V, 0.5 h).
